# Maintaining or Losing Intervention-Induced Health-Related Behavior Change. A Mixed Methods Field Study

**DOI:** 10.3389/fpsyg.2021.688192

**Published:** 2021-06-18

**Authors:** Frida Skarin, Erik Wästlund, Henrik Gustafsson

**Affiliations:** ^1^Department of Social and Psychological Studies, Service Research Center (CTF), Karlstad University, Karlstad, Sweden; ^2^Department of Educational Studies, Faculty of Arts and Social Sciences, Karlstad University, Karlstad, Sweden; ^3^Department of Sport and Social Sciences, Norwegian School of Sport Sciences, Oslo, Norway

**Keywords:** health-related change, motivation, goal achievement, eating behavior, physical activity, intervention-induced change, maintained change

## Abstract

The aim of this mixed methods field study was to gain a better understanding of how psychological factors can contribute to success in intervention-induced behavior change over time. While it can be difficult to change behavior, the use of interventions means that most participants succeed in change during the intervention. However, it is rare for the immediate change to automatically transform into maintained behavior changes. Most research conducted on health-related behavior change interventions contains quantitative studies that investigate key intervention components on a group level. Hence, to bring more knowledge about maintained intervention-induced behavior change, there is need for a study approach that enhances the understanding of individual participants' experiences during and after the intervention. Therefore, the present study, which was conducted in Sweden, used a mixed methods design (triangulation) consisting of pre-, post-, and follow-up quantitative data (questionnaires and body measurements) and qualitative data (interviews), where the individuals' accounts are used to broaden the understanding of the intervention and the behavior change process. All study participants were enrolled in a volitional (fee-based and non-manipulated) intervention given by certified gyms. The quantitative data collection included 22 participants who completed questionnaires and body measurements before and after the intervention, plus 13 complete body measurements 6 months after the intervention. The qualitative data included pre-interviews with 12 participants and six follow-up-interviews. The questions in both questionnaires and interviews related to expectations, efficacy, motivation, goals, achievements, behavior change, and future. Overall, the results show that levels of expectations, efficacy, and motivation cannot be used in isolation to predict maintained intervention-induced behavior change. To successfully extend and maintain immediate change, it was crucial to experience goal achievement (but not BMI change). Furthermore, *enabling talk* was salient in the pre-interviews with participants reporting successful immediate (and maintained) change. By contrast, pre-interview *disabling talk* turned out to be evident in interviews, with participants not responding to follow-up. When the qualitative and quantitative results are summarized and integrated, it appears that subjective goal achievement, combined with enabling self-talk, were crucial factors in successful maintained behavior change.

## Introduction

Health-related behavior change is topical during a time where a pandemic has forced us to take responsibility for our health and make changes accordingly. Physical activity and eating behaviors are fundamental for good health in all individuals. Regular physical activity and healthy eating promote good health and wellbeing and develop resilience against illness (Public Health Agency of Sweden, 2020). Poor physical activity and eating behaviors are linked to obesity (BMI > 30), which brings an increased risk of illness and premature death (Statistics Sweden, 2021). More than two billion people in the world—almost one-third of the entire human race—are obese. In Sweden, which has 10.5 million residents (Statistics Sweden, 2021), the yearly cost for obesity is equal to US$8 billion and is growing. According to a prognosis by The Swedish Institute of Health Economics from 2018, the yearly cost could grow by $2 billion by 2030 (Public Health Agency of Sweden, 2020). To break the downward trend of sedentary lifestyle and unhealthy eating, which has negative consequences for both individuals and societies, policy makers and other stakeholders arrange health-related behavior change interventions. While it can be difficult to change behavior in general, the use of health-related behavior change interventions means that most participants do change their behavior successfully. However, such immediate change experienced during an intervention do not automatically transform into maintained behavior change (Anderson et al., 2001; Curioni and Lourenço, 2005). Therefore, the aim of the present study is to investigate which psychological factors influence the maintenance of immediate behavior change.

Health-related behavior change interventions that aim to help participants implement lifestyle changes that lead to weight loss typically include primarily change of physical activity behavior, changes to eating behavior, and some kind of behavior education or behavior therapy (Khaylis et al., 2010). Learning to change eating behavior includes processes that are both cognitive and automatic. Conscious diet choices, such as adding more healthy foods and reducing amounts and less healthy foods, is an example of a cognitive process competing with automatic responses to stress, or competing goals (such as having the short-term goal of enjoying sweets vs. the longer-term goal of losing weight) (Marteau et al., 2012). Learning to change physical activity behavior might be less multifaceted, since implementing a physical activity routine could be something to manage just once a day. Regardless, maintaining change is complex and previous reviews on maintained intervention-induced behavior change (Avenell et al., 2004; Curioni and Lourenço, 2005; Greaves et al., 2011) have shown that the best maintained weight loss results come from interventions focusing on physical activity and healthy eating in combination, as opposed to one at a time. In studies that focus on important intervention components, recurring factors for successful maintained intervention-induced behavior change include motivation, expectations, and goal achievement (Elfhag and Rössner, 2005; Teixeira et al., 2015).

### Maintenance

The concept of maintenance has been used to refer both to the maintenance of a newly adopted behavior (change) and to the maintenance of an established behavior (habit). However, intervention studies of behaviors like weight control normally focus on maintenance of change rather than on maintenance of individuals who already hold a healthy weight. Physical activity intervention studies usually operationalize successful intervention-induced maintained behavior as engaging in regular physical activity for at least 6 months after the intervention (Dunn et al., 1999). A recent review by Nordmo et al. (2020) suggested that the time-span for maintenance is longer, since the average weight regain back to pre-intervention weight was reached at about 4 years post-intervention. In the present paper, the concept of maintenance will be used to describe the sustenance of immediate behavior change resulting from the participation in a behavior change intervention, which means that intervention-induced behavior change has continued after the intervention. Further follow-ups are needed to tell what happens years forward.

Although the primary goal for both intervention participants and intervention originators is usually behavior change that maintains, rather than immediate change that fades, the latter has been more frequently investigated than the former (Fjeldsoe et al., 2011; Kwasnicka et al., 2016). Recent reviews that have investigated intervention-induced maintained change have highlighted goal setting, an autonomy supportive approach (Samdal et al., 2017), and a clear plan for maintenance (Greaves et al., 2011) as key factors for maintained change. Greaves et al. (2011) conducted a systematic review of reviews on intervention components associated with increased effectiveness in physical activity and healthy eating interventions. This meta-review included 30 reviews and showed maintained behavior change results regarding both physical activity (30–60 min/week of moderate activity at 12–18 months) and weight loss (3–5 kg at 12 months). The intervention effectiveness was increased by targeting both physical activity and eating behavior and using well-established behavior change techniques. Samdal et al. (2017) conducted a systematic review of effective behavior change techniques for physical activity and healthy eating, including the perspective of maintained change. The review included 32 long-term reports and showed that goal setting and self-monitoring through the use of step counters predicted maintained changed behavior. While that type of research shows, on a group level, what type of intervention components are better to include in a health-related behavior change intervention, it does not further our understanding of the individual experiences of a behavior change journey. As proposed in previous research (see, for instance, Greaves et al., 2011), it is important to investigate the underlying process and causality of all proposed behavior change models. Therefore, in addition to comparing interventions and intervention components, it is necessary to hone in on the intervention participants in order to optimize the opportunity for each individual to succeed in changing and maintaining a healthy behavior.

### Goal Setting and Achievement

A fundamental part of changing and maintaining a healthy behavior is the setting and achieving of goals. Locke (1968) introduced the Goal Setting Theory, which shows that setting specific (difficult) goals leads to better performance than setting general (easy) goals. Locke and Latham (1994) developed the Goal Setting Theory, presenting five goal-setting principles that improve the chances for better performance: clarity, challenge, commitment, feedback, and task complexity. Setting a specific goal that includes these principles provides greater clarity, which makes it possible to better notice progress and achievements, and thus monitor and measure change. An example of general (outcome) goal could be to lose weight, while an example of specific (behavior change) goal might be walking for an hour three times a week.

Since the original goal theories, more recent research has found that different types of goals led to different behavior and affective consequences (Deci and Ryan, 2000). Central goal types to make a distinction between are behavior change goals (also called learning goals) vs. outcome goals (also called performance goals) (Dweck, 1986) and approach goals vs. avoidance goals (Carver and Scheier, 1998). Approach goals entail that behavior is driven toward a desired stimulus, while avoidance goals entail that behavior is driven away from an undesired stimulus to avoid negative consequences (Wimmer et al., 2018). Behavior change goals involve implementing a new activity, while outcome goals include a desire to achieve a result. Research shows that people who set outcome goals are more likely to interpret negative outcomes or obstacles to a lack in their own ability, which tends to weaken their continuous effort, leading to repeated adversity. By contrast, people who set behavior change goals tend to interpret negative outcomes or obstacles as a reason for increased effort or the need to modify a used strategy, which tends to lead to improved outcomes (Ames, 1984). Focusing on progress rather than outcome increases the likelihood of improving and maintaining effective strategies when facing obstacles (Bandura and Schunk, 1981). Therefore, in order to successfully change behavior, it is preferable to set specific goals related to the desired activity.

In addition to the configuration of the behavior change goals, the individual's expectations regarding goal achievement are paramount for successfully changing behavior. According to Bandura (1997), expectations can be divided into two types: self-efficacy and outcome expectancy. Self-efficacy is an individual's belief in their own ability to perform a certain behavior (Bandura, 1997). Locke and Latham (2002) argued that self-efficacy is important for goal setting and goal achievement in several ways: people high in self-efficacy generally set higher goals, are more committed to the goals, find better strategies to achieve them, and respond better to negative feedback than those with low self-efficacy. It has also been argued (Byrne, 2002; Elfhag and Rössner, 2005) that self-efficacy is an important factor for maintained behavior change. Outcome expectancy, on the other hand, relates to an individual's belief that a certain behavior will actually lead to a certain outcome. Hence, outcome expectancy affects the anticipated result of performing the desired activity. In short, self-efficacy is belief in one's own ability and outcome efficacy is belief in the behavior leading to the desired outcome. On an overall level, both of these aspects of expectancy relate to an expectation of actually achieving a desired outcome, which has been described as an individual's level of goal expectancy.

The early perspective of goal-directed behavior, including the Goal Setting Theory from the 1960s, which focused on goal-related efficacy as a drive for motivating behavior, has now evolved to include psychological needs. Deci and Ryan (2000) argued that to fully understand goal-directed behavior it is necessary to include psychological needs. They clarified that there is a psychological process, including psychological development and wellbeing behind goal setting, which influences goal pursuit. These psychological needs are cornerstones of Self Determination Theory (SDT), which is a macro-theory of human motivation, development, and health (Deci and Ryan, 1985). For a behavior to occur, motivation is needed. In everyday language, motivation is often referred to in terms of level; thus, high vs. low motivation. However, Deci and Ryan (2008) emphasized the distinction between motivation level and motivation type, showing that type of motivation is more important than amount of motivation for predicting many important outcomes, such as wellbeing and effective performance. According to Deci and Ryan (2008), the most central distinction in SDT is between autonomous and controlled motivation. Paying money to participate might be the most highly volitional form of intervention participation. However, paying money to participate might also influence participants' expectations. Thus, in addition to investigating participants' levels of motivation in terms of their goals and experienced efficacy, it is necessary to look into their types of motivation (Deci and Ryan, 2008).

### Research Gap

In order to better understand how intervention-induced immediate behavior change can be maintained, it is necessary to include longer-term post-intervention follow-up measurements, as opposed to only immediate post-intervention measurements (Whitt-Glover et al., 2014). Additionally, collecting field-data in a non-influenced real-world setting leads to a higher ecological validity. Since most previous research has focused on group-level findings on intervention components (e.g., Greaves et al., 2011; Samdal et al., 2017), it is essential to include intervention-experience data on individuals' level. In order to gain a better understanding of which psychological factors affect an individual's chances of maintaining an intervention-induced behavior change, it is necessary to gain a deeper understanding of the individual's experience of participating. This is only possible by combining qualitative and quantitative data. Mixed methods design is both a methodology and method that mixes quantitative and qualitative approaches in data collection and analysis, and interprets the results from the analyses together. Collecting the quantitative and qualitative data at the same phase, giving them equal importance and interpreting them simultaneously is called a convergent parallel mixed method (Creswell and Plano Clark, 2011) and can be used to build understanding by presenting interview stories (narrative data) complementing links and numbers of statistical data (Creswell, 2013).

In sum, we argue that, to follow previous directions of future studies on maintained behavior change, interpreting quantitative links together with qualitative narratives from post- and follow-up measurements are required to create a better understanding of each individual's experience. In so doing, we are able to investigate how to optimize each individual's intervention journey to succeed in desired maintained changed behavior.

Against this background, the aim of the present study was to answer the following research questions:

RQ1: Do intervention participants' levels of expectations, efficacy, and motivation predict intervention-induced immediate and maintained behavior change?
RQ2: How do intervention participants' perceptions describe their expectations, efficacy, motivation, and goals in relation to intervention-induced immediate and maintained behavior change?

## Study Overview

Against the backdrop of research showing that intervention-participants given incentives are not as autonomously motivated to maintain the intervention-induced change as volitionally participating individuals (Deci et al., 1999; Gneezy et al., 2011; Arien et al., 2012), the present study was conducted with the aim of including participants who were not persuaded by any incentives whatsoever. Hence, the present study only includes participants who volitionally engaged in the intervention and even invested their own money by paying a participation fee.

### Methods

Both data collections were based on a 3-month lifestyle change intervention given by certified gyms where participants were recruited for the study. The study was conducted on a current intervention concept based on the typical lifestyle change program foundation: eating and physical activity inspiration, education and individual plans including goal setting, tools, and monitoring/results check-ups. The intervention groups consisted of six participants and were directed by licensed personal trainers certified in the intervention concept. The concept included two small-group personal training (PT) sessions per week, recurrent group-coaching sessions each week, diet advice, and recipes. The enrollment fee was equivalent to USD 650 for these 3 months of combined program. The monthly cost (650/3 = 216) can be compared to the monthly cost of an offer at one of Sweden's most commonly used gym, including a similar amount of small group PT sessions on weight loss but without the other features. Gym fees (entrance only) differ widely between gyms and subscription time, but an average range is between 36 and 72 USD per month on a 12-month subscription (Gym Guide, 2021). One PT session costs about 120 USD.

The participants in the present study vary in physical activity background, but a recurring factor is no regular engagement in physical activity before the intervention. Maintenance of change—that is, when participants sustained the intervention-induced behavior more than 6 months—is referred to as maintained or long-term change (of eating and physical activity behaviors). Due to availability, the quantitative data collection and the qualitative data collection were made on different participants, but all were participants in the same lifestyle change program. All interviews were conducted by the same researcher (FS) to maintain consistency between the interviews.

The type of mixed methods design used in present study is triangulation (also referred to as convergent or concurrent nested mixed methods design). Data for the quantitative and qualitative studies were collected concurrently and analyzed after both collections were completed. Post-measurements were used to measure intervention-induced immediate behavior change, while follow-up measured maintained change. See Table 1 for study overview intervention timeline.

**Table 1:** Study overview intervention timeline.

	**Pre**	**Post**	**Follow-up**
Quantitative data collection	Questionnaire + body measurements	Questionnaire + body measurements	Questionnaire + body measurements
Qualitative data collection	Interview		Interview

## The Quantitative Data Collection and Results

To investigate intervention component links and importance for change, the quantitative data collection was conducted by contacting gyms offering the current behavior change intervention and starting to recruit participants to the study. We chose to collect data from three gyms in two cities in Sweden, with the aim of widening the perspective from investigating just a specific gym depending on only a few personal trainers.

### Methods

#### Procedure

At the first day of the intervention, 39 participants were asked to voluntarily enroll in the study by filling out a consent form. The participants (*n* = 38) then answered pre-questionnaires about expectations, efficacy, motivation, and goals. On the last day of the intervention, the participants filled out post-questionnaires with questions about the same components as in the pre-questionnaire, as well as about goal achievement and experiences during the intervention. Additionally, the participants approved the researchers collecting (de-identified) body measurements data measured by the personal trainer at the gym before and after the intervention, as well as at a follow-up 6 months after the intervention.

#### Participants

**Figure 1:**
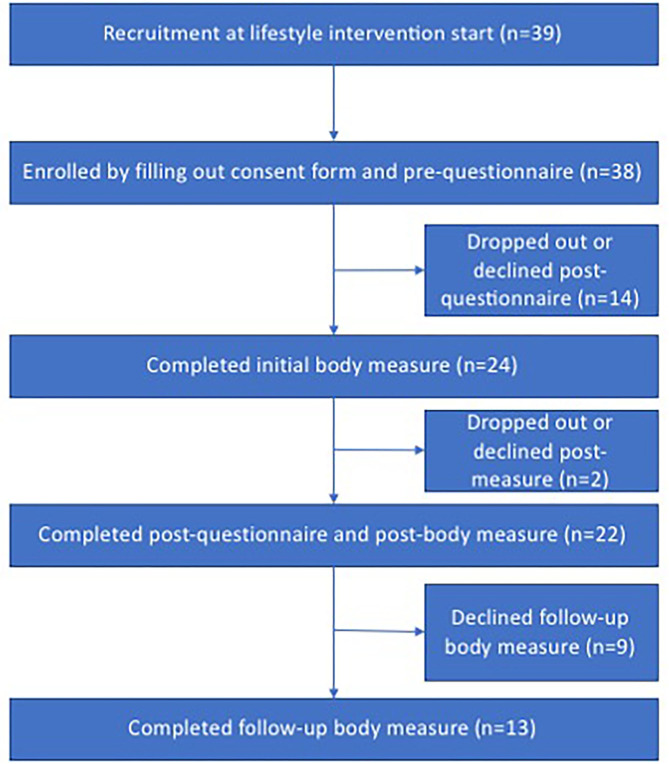
Flowchart recruitment for quantitative data collection.

The data collection resulted in 22 complete pre- and post-questionnaires (100 percent female, age: *M* = 50, Range = 28–71, SD =11.8) and 13 participants participated in the follow-up body measurements; see Figure 1 for recruitment and drop-out details. The average BMI of the participants was 29.91 (SD = 2.98), ranging from 25.78 to 37.57. The response rate for all measures was 33.3 percent.

#### Material

The pre-questionnaires included efficacy level, level of goal expectancy, and motivation level. In order to minimize the effort needed to participate in the study, the data collection was conducted with single-item measures as much as possible. The validity of single-item scales has been shown to be satisfactory in general (Bergkvist and Rossiter, 2007, 2009). More specifically, single-item scales have been used to measure self-efficacy (e.g., Hoeppner et al., 2011), expectancy (e.g., Ilgen et al., 1981; Wanous et al., 1997), and motivation (Skarin and Wästlund, 2020). The measurements are presented in further detail below.

##### Efficacy Level

We used *efficacy*, a merged measure of self-efficacy and outcome efficacy, to measure the participants' overall belief in their own ability to change behavior and their belief that the changed behavior would lead to their desired outcome. The efficacy measure was calculated by means of answers to the following questions: “Regarding the overall intervention, how strong is your belief that you have the ability to succeed in reaching your goals?” (self-efficacy) and “Regarding the overall intervention, how strong is your belief that the intervention content and design will help you reach your goals?” (outcome efficacy). Both questions used a seven-point Likert scale ranging from 1 (“not at all”) to 7 (“fully”).

##### Goal Expectancy

Given that previous research regarding expectancy is diverse in both the conceptualization and focus constructs (Klein, 1991), we chose to complement the pre-measurements with goal expectancy, with the aim of capturing the participants' expectancy of actually reaching their specific goals. While one can have a general belief in their own ability, as well as in the intervention design, life circumstances play a role when it comes to expectancy in reaching specific goals. Therefore, we asked the participants to report their belief in reaching their specific goals of eating, weight, and other (the latter was intended to cover individual goals of various types). These goal expectancies were each measured with seven-point Likert-scale questions— “How well do you think you will reach your goals regarding eating?” “How well do you think you will reach your goals regarding weight?” and “How well do you think you will reach your goals regarding what you reported above as other goals?” —ranging from 1 (“not at all”) to 7 (“fully”). We measured total goal expectancy based on these expectancies.

##### Motivation Level

In line with Markland and Hardy (1997), motivation was measured to indicate the participants' level of motivation to accomplish the intervention. Before the intervention, motivation level was measured with the question “*How motivated are you to implement the intervention?*” on a seven-point Likert Scale ranging from 1 (“not at all”) to 7 (“very motivated”).

The post-questionnaires included motivation type, goal achievement, and BMI change compared to before the intervention.

##### Motivation Type

In line with Deci and Ryan (2008), after the intervention the participants were asked to report their motivation type during the intervention. In order to obtain a clear outline of motivation type, in relation to each other, we wanted to take both goal-directed behavior (Locke and Latham, 2002) and autonomous vs. controlled motivation into consideration (Deci and Ryan, 2000). Therefore, we chose to collect the motivation reports as ratio of motivation orientation between doing it for a fun experience and doing it to reach goals, measured by the question “*Now in retrospect, what motivated you to accomplish the intervention?*” on a seven-point Likert Scale ranging from 1 (“fun experience”) to 7 (“reach goals”).

##### BMI Change

Body mass index (BMI) is an objective measurement of bodily change, which should not be used to assess detailed body composition (Beechy et al., 2012), but is a commonly used measurement of weight loss used in many weight-loss studies. BMI (calculated by body weight in kilos divided by body height in meters squared, or kg/m^2^) was measured before and after the intervention. BMI change was calculated by subtracting the post-measurement BMI from the pre-measurement BMI.

##### Goal Achievement

In a meta-analysis from 2016 (Harkin et al., 2016), significant effect sizes for monitoring goal progress were shown across a range of behavior outcomes. In addition, the amount of research showing that goal-focused outcome measures support reliability, validity, and clinical utility is growing (see, for instance, Elliott et al., 2016; Sales and Alves, 2016). Further, previous research has suggested using more idiographic tools when measuring goal achievement, such as including self-reporting (Lloyd et al., 2019). In the present study, the personal trainers instructed the participants to set eating behavior goals, physical activity goals, weight goals, and other goals before the intervention started. Post-intervention, the participants were asked to report their goal achievement regarding each of these goals (eating, physical activity, weight, and other) on a seven-point Likert Scale ranging from 1 (“not at all”) to 7 (“fully”). Overall goal achievement used in the correlational analysis was calculated by means of these four goal achievement reports and is thus the participants' self-reported experience of intervention-induced immediate behavior change.

The follow-up data collection was conducted to measure maintained change.

##### Maintained Change

When referring to successful maintenance, intervention studies usually operationalize with the criterion of engaging in regular physical activity for at least 6 months after the intervention (Dunn et al., 1999). Six months after the interventions ended, the personal trainers invited the participants to a free follow-up full body measurement identical to the pre-intervention body measurement they had all had. Comparing post-intervention body weight in kilos (that is, at the end of the intervention) and follow-up body weight with the pre-intervention body weight provides objective measurements of the results of the participants' immediate and maintained results of behavior change.

### Results From Quantitative Data Collection

In order to answer Research Question 1 (“Do participants' levels of expectations and motivational factors predict immediate and maintained behavior change?”), a Spearman's rho correlational analysis was conducted to clarify the levels of expectation and motivational factors' connection to immediate and maintained change, as well as their relationships, respectively (see Table 2 for descriptive results). Additionally, a Mann-Whitney U Test was conducted to investigate which specific goal achievement (physical activity, eating behavior, weight, or other), if any, is associated with maintained change. All statistical analysis was conducted with IBM SPSS Statistics for Windows, Version 21.0.

**Table 2:** Means (M) and Standard deviations (SD) for variables included in the analysis.

	**N**	**M**	**SD**
Goal expectancy	22	6.09	0.78
Efficacy level	22	5.95	0.89
Motivation level	22	6.45	0.91
Motivation type	22	5.77	1.34
Goal achieve	22	5.05	1.12
BMI change	22	−2.67	1.40
Maintained change	22	1.73	0.83

#### Correlations

**Figure 2:**
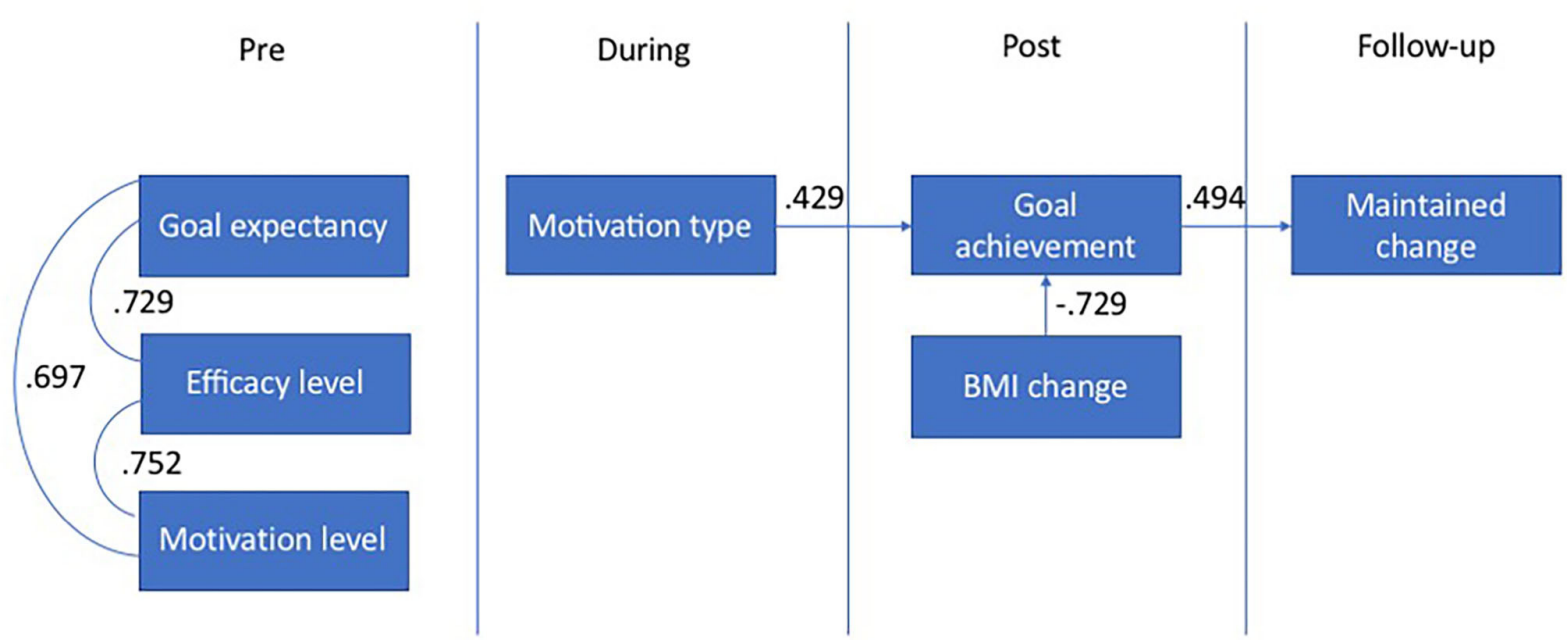
Intervention timeline correlations.

To illustrate relationships and chronology, a timeline of correlations (Figure 2) and a correlations table (Table 3) are presented below. The correlational analysis shows that the three factors measured before the intervention—levels of goal expectancy, efficacy, and motivation—are strongly correlated. Surprisingly, these three factors did not correlate with any other factors measured later: motivation type, goal achievement, BMI change, or maintained change. The correlational analysis also shows that displaying a higher post-BMI-drop was, quite expectedly, correlated with higher goal achievement. Motivation type and goal achievement were correlated (that is, the more goal-orientated, the more goal achievement), and goal achievement was correlated with maintained change.

**Table 3:** Correlation measurements (Spearman's rho).

		**Goal expectancy**	**Efficacy level**	**Motivation level**	**Motivation type**	**Goal achieve**	**BMI change**	**Maintained change**
Goal expectancy	Correlation Coefficient	1	0.729^**^	0.697^**^	0.265	−0.037	−0.04	−0.002
	Sig. (2-tailed)	.	0	0	0.234	0.871	0.861	0.992
	N	22	22	22	22	22	22	22
Efficacy level	Correlation Coefficient	0.729^**^	1	0.752^**^	0.355	0.008	−0.151	0.016
	Sig. (2-tailed)	0	.	0	0.105	0.97	0.501	0.942
	N	22	22	22	22	22	22	22
Motivation level	Correlation Coefficient	0.697^**^	0.752^**^	1	0.373	0.137	−0.321	−0.147
	Sig. (2-tailed)	0	0	.	0.087	0.545	0.145	0.514
	N	22	22	22	22	22	22	22
Motivation type	Correlation Coefficient	0.265	0.355	0.373	1	0.429^*^	−0.412	−0.081
	Sig. (2-tailed)	0.234	0.105	0.087	.	0.041	0.05	0.713
	N	22	22	22	23	23	23	23
Goal achieve	Correlation Coefficient	−0.037	0.008	0.137	0.429^*^	1	−0.729^**^	0.494^*^
	Sig. (2-tailed)	0.871	0.97	0.545	0.041	.	0	0.017
	N	22	22	22	23	23	23	23
BMI change	Correlation Coefficient	−0.04	−0.151	−0.321	−0.412	−0.729^**^	1	−0.322
	Sig. (2-tailed)	0.861	0.501	0.145	0.05	0	.	0.134
	N	22	22	22	23	23	23	23
Maintained change	Correlation Coefficient	−0.002	0.016	−0.147	−0.081	0.494^*^	−0.322	1
	Sig. (2-tailed)	0.992	0.942	0.514	0.713	0.017	0.134	.
	N	22	22	22	23	23	23	23

** *Correlation is significant at the 0.01 level (2-tailed)*.

* *Correlation is significant at the 0.05 level (2-tailed)*.

#### Mann-Whitney U Test

An independent samples Mann-Whitney U Test was conducted to compare the participants who participated in the follow-up measurements with those who chose not to do so, regarding the four measures of goal achievement (physical activity behavior, eating behavior, weight, and other). The results show that those participants who responded to follow-up measurements reported a significantly (*U* = 98.5, *p* =0.036) higher level of goal achievement regarding physical activity than those who did not respond. With regard to eating behavior, the results showed that those who responded reported almost significant (*U* = 96.5, *p* = 0.055) higher levels of goal achievement than those who did not respond. While the reported levels of goal achievement regarding weight loss and other goals were not significant (ps > 0.05), they followed the same pattern in that those who responded for follow-up measurement reported higher levels than those who did not respond (see Table 4 for M and SD).

**Table 4:** Means (M) and Standard deviations (SD) comparing participants who participated in the follow-up with participants who did not.

**Goal completion**	**Follow up (Yes/No)**	**N**	**M**	**SD**
PhysActivity	No	11	5.18	0.98
	Yes	12	6.08	1.00
Eating	No	11	4.27	1.68
	Yes	12	5.58	1.38
Weight	No	11	4.27	1.79
	Yes	12	5.08	1.38
Other	No	11	4.82	1.33
	Yes	12	5.50	0.80

### Quantitative Discussion

The pre-measurements of goal expectancy, efficacy, and motivation levels correlate with each other respectively, but do not predict either immediate or maintained behavior change. To successfully extend immediate change into maintained change, experiencing goal achievement (but not BMI-change) is crucial. This means that to succeed in maintained behavior change, the experience of goal achievement during the intervention is more important than outcomes the goal achievement produces (weight loss). The results of the Mann-Whitney U Test on goal achievement categories clarify that the most important goals to achieve during the intervention are behavior change goals such as learning new physical activity behavior- and eating behavior routines. Thus, experiencing the achievement of outcome goals such as weight loss is less important. In sum, both outcome goals and outcome results are less important than behavior change goals when aiming to succeed in intervention-induced maintained change. These are intriguing findings since behavior change is usually desired because of its consequences or outcomes. Focusing too much on the intervention-induced immediate outcomes will not help maintain behavior change, which means that desired maintained outcomes will remain absent. Therefore, focusing on setting behavior change goals such as physical activity behaviors and eating behaviors, and achieving these behavior change goals, are essential for maintaining intervention-induced behaviors and their outcomes. A noteworthy question that remains after the quantitative analysis is why the pre-measurements are highly related to each other, but do not seem to be related to the post-measurements regarding the effects of the intervention.

## The Qualitative Data Collection and Analysis

Previous research in the area is dominated by quantitative studies investigating which intervention components result in the biggest success. Our aim is to understand more about the process of each individual's intervention journey in order to be able to support each one to maintain intervention-induced behavior change. From choosing the focus of understanding the individuals' experiences to be able to suggest better ways of support, the mixed method design includes a qualitative data collection conducted in the form of interviews, which complements the quantitative data collection presented above. The presentation of the qualitative data collection and analyses are divided into two parts: the pre-interview and the follow-up interview parts. For both pre-interviews and follow-up interviews, the analysis of the transcribed data was conducted by following the six phases of thematic analysis (Braun and Clarke, 2006). The qualitative software used was NVivo (version 12.2.0.443), which helped keep the data organized and made the process of coding and analysis more transparent and easier to replicate. All interviews were conducted by the primary investigator, who conducted the initial coding and categorization of themes. The second and third authors then reviewed the categorization and acted as “critical friends,” asking questions during the analysis and promoting alternative explanations and interpretations of the data (Marshall and Rossman, 2006).

### Method

#### Procedure

At the first day of the intervention, 12 participants were asked to voluntarily enroll in the study by filling out a consent form. The participants (*n* = 12) were then booked for pre-interviews about expectations, efficacy, motivation, and goals. The pre-interviews were held within the first 2 weeks of the intervention. Six months after the intervention, the participants were contacted again to book date and time for follow-up interviews.

#### Participants

**Figure 3:**
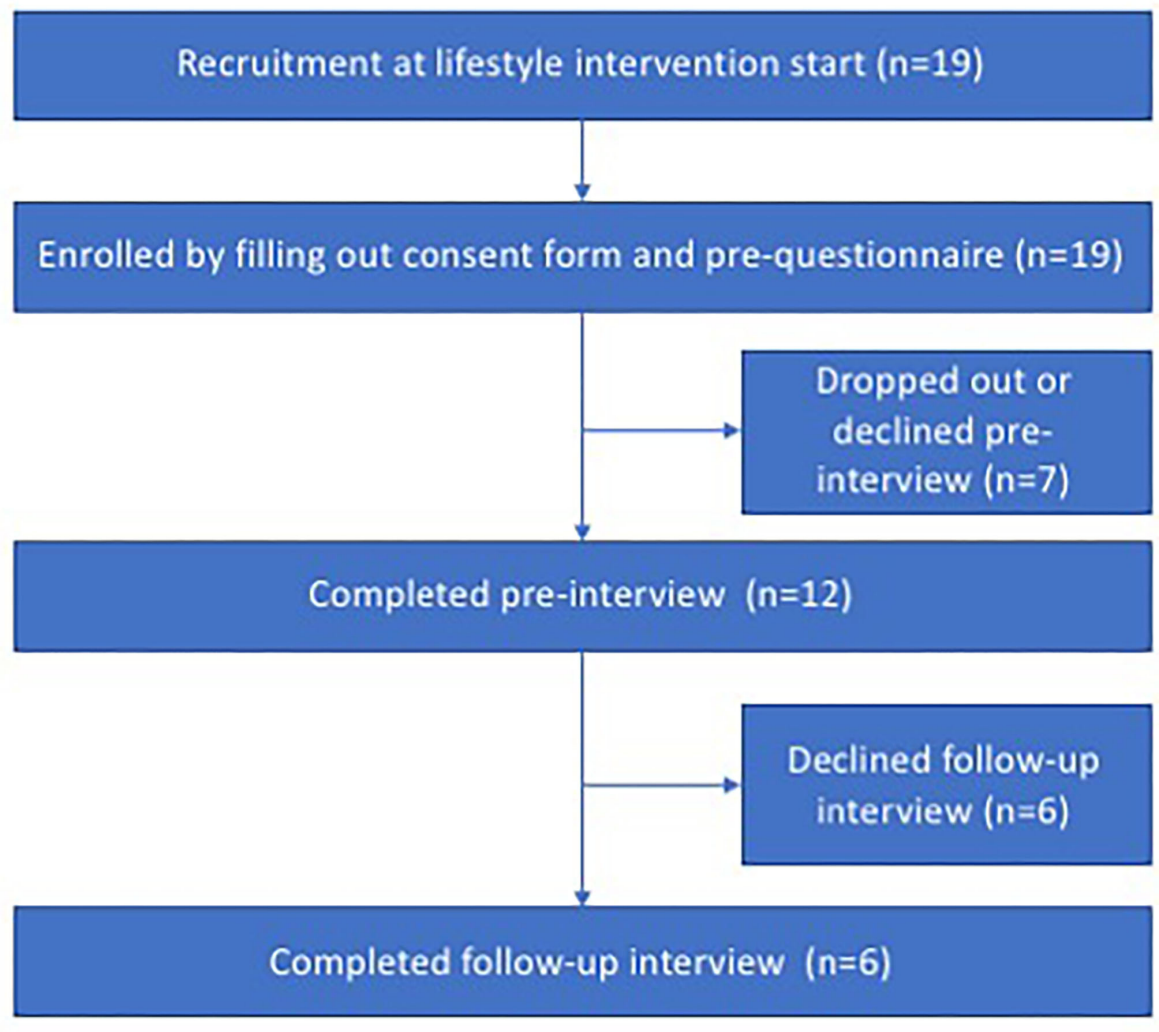
Flowchart recruitment qualitative data collection.

Note: Two posts were eliminated due to medical issues, see section Analysis of Pre-interviews.

The qualitative data analysis entailed 10 (one male and nine female) complete initial interviews, and five (one male and four female) responses to the follow-up interviews six months after the intervention (see Figure 3). One participant who did not reply to the follow-up reported that she dropped out from the intervention itself due to lack of time. The other four participants who did not reply to follow-up did not report a reason; instead they made themselves unavailable. The interviews were held at places that suited the participants; either at their workplace, at the university or at a public café or library.

#### Material

The interviews were based on the same topics as the questionnaires in the quantitative part. Thus, the pre-interviews included questions about motivation, efficacy, expectancy, and goals, and the follow-up interviews included questions about motivation, goal achievement, BMI change, and health-related behavior change. The open-ended nature of interviews gave the participants opportunities to express themselves in their own words, which provided a broader and deeper representation of their answers. The pre-interview was divided into two sections, starting with a brief history of the participant and reasons for participating in the intervention, moving on to current eating behavior and physical activity behavior, goals, and expectations about participating in the intervention. The follow-up was divided to first focus on goal achievement (that is, eating and physical activity behavior today), then moving on to success factors, motivation type, and eventual goals for the future.

### Pre-interviews

The data analysis, discussion, and results from the pre-interviews are presented below.

#### Analysis of Pre-interviews

The aim of the pre-interviews was to collect narratives that could be utilized to increase our understanding of intervention-induced immediate and maintained behavior change. Hence, in order to answer Research Question 2 (“Do participants' narratives regarding expectations and motivational factors predict immediate and maintained behavior change?”), pre-interview data was collected and thematized, and is presented in a form that reveals the flavor of the discussion and defines the essence of distinctions in discussions between the main themes. The themes that arose through analysis of the pre-interviews are presented below, followed by a description of the analysis process.

The first phase of thematic analysis involves familiarizing oneself with the data (Braun and Clarke, 2006), which was done by entering the transcript interviews into NVivo and reading through them carefully several times to assemble an initial list of ideas about what is especially interesting. In Phase 2, the first codes were created from concepts we found appropriate to describe patterns and noteworthy parts of the data at that point. This led to 30 nodes filled with extracts of data. In Phase 3, we started searching for themes among the codes. A few similar codes were merged into the same, and a few others were eliminated because they were beyond the scope of the research question (for example, what made the participants overweight). The remaining codes were organized into three candidate themes (negative talk, positive talk, and success factors). In Phase 4, we reviewed the themes in order to refine them and condensed them into themes that represented the data set properly and were in line with the research question. In the second level of phase four, we reread the entire dataset and decided to subtract two posts because they did not “accurately reflect the meanings evident in the data set as a whole” (Braun and Clarke, 2006). Thus, two data posts were eliminated due to medical issues, since they would have skewed the picture of intervention participation.

**Figure 4:**
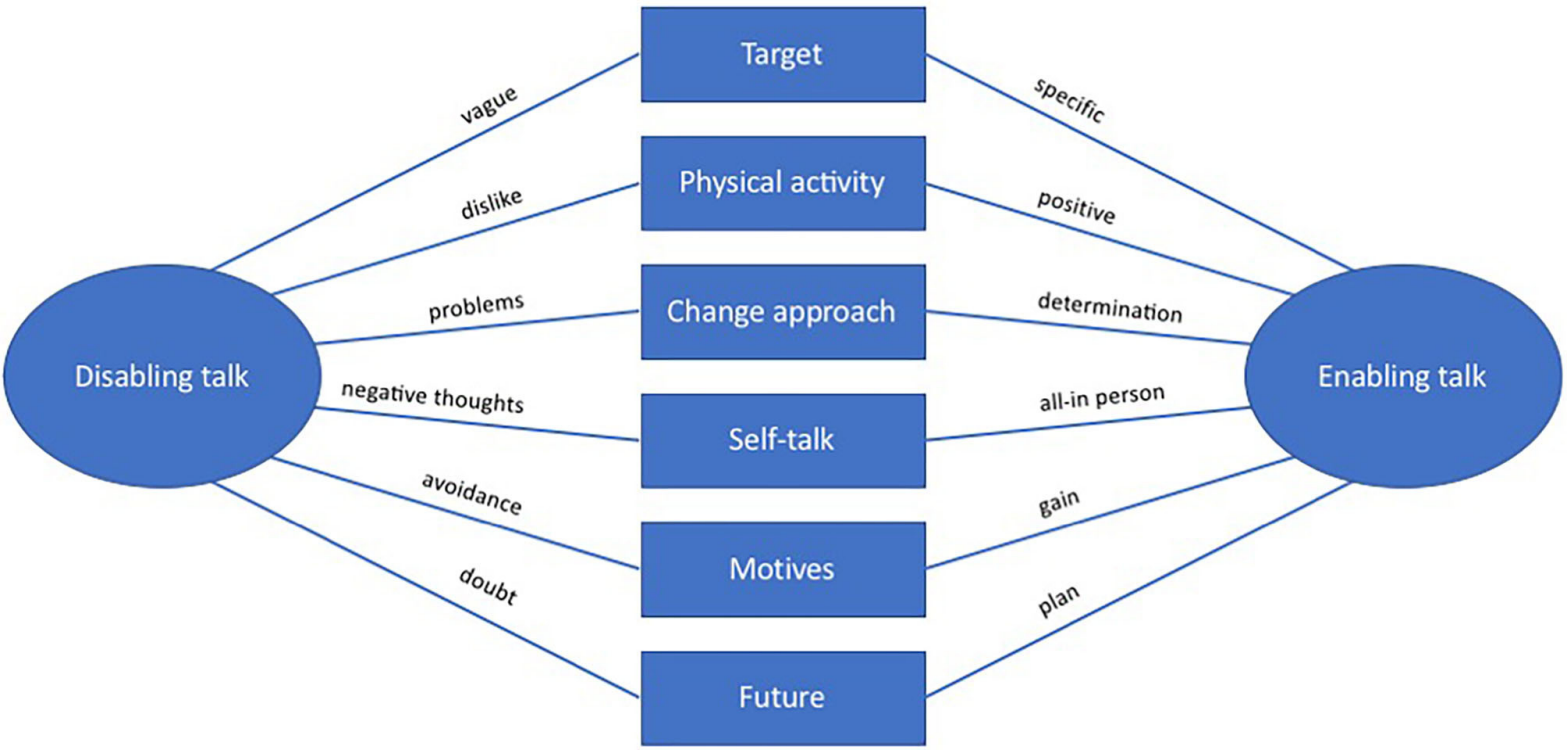
Final thematic map of pre-interviews.

In Phase 5 we wanted to identify the essence of each theme and the stories they tell, in line with Braun and Clarke (2006). This defining and refining resulted in *success factors* being merged into *positive talk*, since those were identical. *Negative* and *positive talk* were then renamed as *disabling* and *enabling talk*, which became the final main themes. In Phase 6 the themes were described verbally, writing the report in connection with the thematic map. See Figure 4 for the final thematic map.

**Figure 5:**
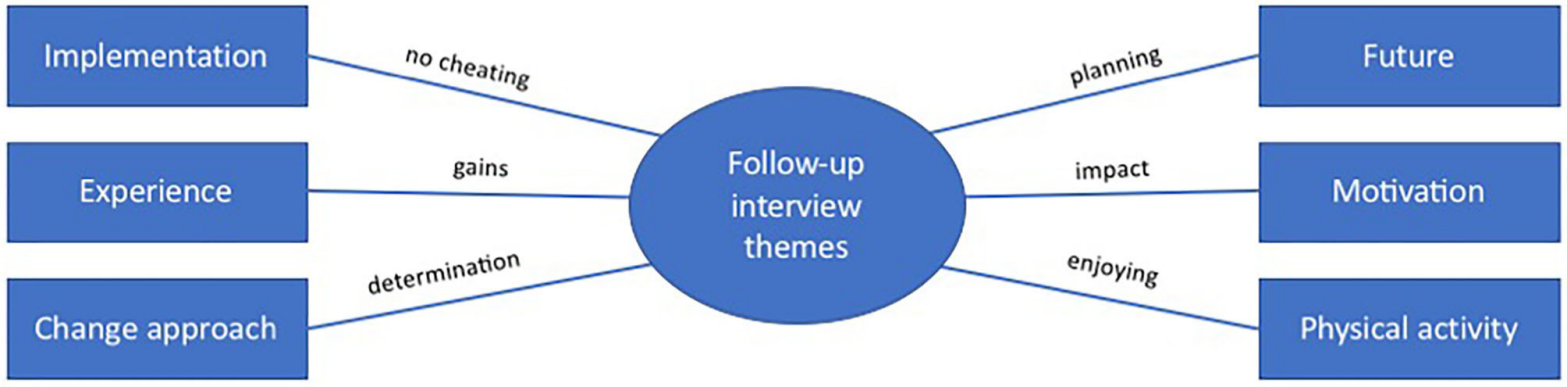
Final thematic map of follow-up interviews.

To describe the content of Figure 5, Table 5 provides definitions of each sub-theme and short description of the characteristics for both disabling talk and enabling talk on each sub-theme.

**Table 5:** Defintions of sub-themes and their main themes characteristics.

**Sub-theme**	**Definition**	**Disabling talk characteristics**	**Enabling talk characteristics**
Target	Reported reasons for joining the intervention	Difficulty defining a clear target	Refer to this as a specific time where change seems especially necessary and possible
Physical activity	Spontaneous reflection about physical activity	Express dislike and anxiety	Express enjoyment and liking
Change approach	Spontaneous reflections about change	Problems and how hard it is to change	Change is about determination and creating routines
Self-talk	Spontaneous reference to what kind of person they are	Recurring talk about negative thoughts	Refer to oneself as an all-in person
Motives	Reasons for wanting change	Avoidance	Gain
Future	What is your plan for the future to successfully maintain change?	Express doubt or difficulty to change and maintain change	Plan for maintaining new behaviors and managing upcoming obstacles

For each sub-theme, there is disabling talk and enabling talk, which are described in more detail with descriptive extracts from the interviews below.

##### Target

When answering questions about reasons for engaging in the intervention, talk characterized by difficulty in defining a clear target for participating in the intervention or why this would be a better time than any other was categorized as disabling talk. Descriptive quotes for such vagueness include: “*Well, no, I… No, I don't know, it's hard. I don't know”*.

Talk characterized by clear reasons and this period of life as a specific time where this change seems especially necessary and possible was categorized as enabling talk. Descriptive quotes include: “*It's just now I feel I have the strength and the support to make it”*.

##### Physical Activity

During the interview, the participants came to talk about physical activity, since that was one of the main behavioral changes that the intervention aimed to help them with. This topic opened the way for spontaneous expressions about whether they liked it or not. Talk characterized by a dislike for physical activity or the gym was categorized as disabling talk. Defining quotes included: “*I don't like it [physical activity]”*.

Talk characterized by positive emotions regarding physical activity was categorized as enabling talk. The positive words about physical activity were very pronounced and clear in these interviews. Examples include: “*I can notice right after a work-out that I get more energy, right away. So it's very instant”*.

##### Change Approach

The change perspective of engaging in an interview clearly opened up the participants to talk spontaneously about their approach regarding the bigger picture of change. Talk characterized by failure and the difficulty of changing were categorized as disabling talk. Descriptive quotes include: “*I've tried by myself and failed over and over again”*.

Talk characterized by arguments for change as a product of planning, priority, and determination to actually change lifestyle behaviors was categorized as enabling talk. An example quote is: “*That's it really, that I just have to want to”*.

##### Self-Talk

During the interviews, the participants spoke spontaneously about what kind of person they are and how they talk to themselves. Talk characterized by how negative thoughts hinder behavior change, how negative thoughts are used to punish oneself, and an exhausted wish to change these negative thoughts was categorized as disabling talk. An example quote is: “*I get stuck in negative thought paths that makes me end up thinking” “why should I go to the gym?”*

Talk characterized by descriptions of oneself as an “all-in person” who commits fully to the things they decide to do was categorized as enabling talk. A descriptive quote for this category include: “*I'm like when I've decided something, then I go all-in so to speak”*.

##### Motives

Answers to the question “Why do you want change?” were dominated by avoiding something (such as disease or early death), and were categorized as disabling talk. An example quote is: “*If I don't lose weight, in a couple of years I will have diabetes, I know that. I have severe risk for it”*.

Answers dominated by gaining something (positive feelings, alertness, strength) were categorized as enabling talk. A descriptive quote is: “*… It made a lot for alertness and mood* [when cycle commuting to work], *that I had more energy even when I got home even if I got temporarily tired legs, I got a lot of energy out of it. And that's another strong reason that I'm doing this, that I want to be more alert basically, more energized and more alert”*.

##### Future

When talking about the future, including planning for maintenance of intervention-induced behaviors, the participants reflected spontaneously on this topic. Comments that were characterized by doubt and that continuing physical activity and new eating behaviors in the future will be difficult were categorized as disabling talk. A descriptive quote included: “*If I had a structured plan, then maybe I'd be able to reach my goal, but now after April I will be on leave and travel around the world and then I know it's really hard to… I won't train, and it will be really hard to eat [well] too”*.

Talk characterized by planning how to reach goals, including how to solve upcoming hindrances, which involves lessons learned from the past and how to overcome soft spots, was categorized as enabling talk. An example quote is: “*When it comes a… [hinder] It's not* ***if****, it's when. But then it will… then I will call my brother again [which works every time], and then just go to the gym and see what happens”*.

#### Results and Discussion Pre-interviews

The two main themes (enabling talk and disabling talk) on each of the six sub-themes describe the essence of how the participants express themselves when being exposed to the same questions about motivation, efficacy, expectancy, goals, and post-intervention plan.

In sum, the themes arising in the pre-interviews were enabling or disabling talk about target, physical activity, change approach, self-talk, motives, and future.

*The disabling talk* category orbited problem orientation and hindrances for change. This talk appears to be flavored by the statement “Why I will fail,” expressing vagueness, problems, and/or negativity. The fail flavor consists of spontaneously expressed doubt and hindrances for change in each specific sub-theme. Examples of the fail flavor in a more general expression are: “*I don't think I will lose that much [weight] within 3 months”* and “*If I'm stressed, even if I've gone to the gym and when I'm done I don't think it mattered. I didn't feel satisfied or that I succeeded, or happier”*.

The *enabling talk* category signal determination, power, and/or structure and orbit solution orientation, and reasons for succeeding in changing behavior, and appear to be flavored by the statement “Why I will succeed” in each specific sub-theme. Some example quotes of spontaneously expressed progress in general, credit to oneself, and things that speak to success regarding changing behavior are: “*I walk around and, in my head, push myself all the time now”* and “*The easiest way to say it is that I am different now. It's me who changed, it's me who has will and drive to make sure it will be a change”*.

### Follow-Up Interviews

This section presents analysis, discussion, and results from the follow-up interviews.

#### Analysis Follow-Up Interviews

The purposes of the follow-up interviews were to follow-up the intervention-induced immediate change and to gather a better understanding of the intervention process as well as the post-intervention process. The follow-up interview analysis was conducted through the same course of action as the thematic pre-interview analysis described above (Braun and Clarke, 2006).

The first phase of familiarization with the data was done by entering the transcript interviews into NVivo and reading them through carefully for an initial list of intriguing matters. The second phase led to 14 nodes filled with data extracts. In the third phase, the codes were placed into sub-themes and, after some merging and elimination, the codes were organized into sub-themes on the most prominent matters. In Phase 4, the sub-themes were refined to six, which represented the data set properly and were in line with the aim of the follow-up interview analysis: to understand the individuals' intervention process in order to provide additional keys for supporting successful intervention-induced goal achievement and maintained change. In Phase 5 we refined the caption of the main theme from *keys for maintained change* to *follow-up interview themes*, which we found better describes the story it tells. In Phase 6, the main theme and sub-themes were described by verbally writing the report in connection with the thematic map (see Figure 5).

#### Results and Discussion Follow-Up Interviews

All follow-up interviews included reports of immediate and maintained behavior change success. The pre-interview enabling talk quotes were all extracted from interviewees reporting maintained change at follow-up. Likewise, all of the disabling talk quotes were extracted from interviewees who did not respond to the follow-up. This meant that those interviewees who did not respond to the follow-up were likely to be less successful at maintaining the intervention-induced behavior change. For all follow-up interviewees, the maintained behavior change led to maintained or continuous weight loss.

The follow-up interview analysis revealed that key factors for being able to maintain intervention-induced change are as follows: not cheating during the intervention, experiencing positive gains with the new behaviors, seeing change as a determination issue, enjoying physical activity, using the intervention-induced impact (with emphasis on the positive emotions the new behaviors bring) as motivation to hold on and continue, and planning food and training. Descriptions of each sub-theme and supporting quotes are provided below.

##### Implementation

The talk among those who successfully maintained change concerned strictness during the intervention, which included not cheating. A descriptive quote is: “*Yeah, I followed the program and I followed it pretty… or, I actually didn't miss a single meeting”*.

##### Experience

Experiencing positive aspects and gains from the intervention-induced behaviors was prominent in the talk for being able to maintain change. Experiencing the intervention-induced eating behavior changed as something that the participants could live with in their everyday life, rather than a sacrifice, and experiencing the intervention-induced physical activity behavior as something that feels so good that the participant did not want to be without it, rather than a burden. Example quotes are: “*No, I don't think it is [a sacrifice to eat diet food], no”; “I get so full that I… yeah, I'm good on it [the diet food]. And that's what makes me believe that I will be able to continue this, that I find food that works okay”;* and “*… this with changing to train more and eat better, I don't think it's been hard, I haven't experienced it as hard at all actually”*.

##### Change Approach

Determination was a key word that arose among conversations about change. Viewing change as a determination issue was important for maintaining intervention-induced change. Illustrative quotes are: “*… when I come to that point, ‘No—Now I'm determined' then I do it”*; and “*Now I have something I need to complete. I can't just quit. That's not me”*.

##### Physical Activity

Enjoyment of physical activity clearly stood out in all of the follow-up interviews, expressed both as pure enjoyment and happiness of finding that source of energy, and for being a key to continuous engagement in physical activity day after day. The fact that enjoying physical activity is a factor in successfully maintaining behavior change means that it can be understood by seeing it as a behavior driven by autonomous motivation, which gives instant internal reward. Enjoying physical activity means that the behavior itself creates positive emotions, which makes it satisfying to continue. Descriptive quotes for enjoying engaging in physical activity include: “*And now I train regularly and it feels very good too, and now I get frustrated if I can't train and that's something I haven't felt before, that's where I wanted to be”;* and “*… That I feel good, that I get happy from it. I feel that I do something that is good for me”*.

##### Motivation

Among talk about motivation to proceed and maintain intervention-induced behavior change, experienced gain from the intervention was prominent. Gains were described as a positive impact on physical and psychological wellbeing from achieving goals, experiencing bodily change, obvious relief of pain and earlier health problems, pride in own accomplishments, and generally feeling better about oneself. Example quotes expressing gains that motivate maintained behavior include: “*I mean, now I have worked for something that I think is worth keeping and I don't want to ruin. And I notice how much better I feel; I'm more satisfied with myself and I'm more alert, have more energy…”* and “*I've lost 20 kilos so I have a lot… no pain in the knees and stuff, can climb stairs again, which I haven't done in many years because it's been hurting and such”*.

##### Future

Discussions about the future highlighted the importance of planning for physical activity and eating. Techniques to maintain the intervention-induced behaviors include deciding before a meal to only have one portion instead of two, planning dinner ahead to avoid energy level influencing the decision in the moment, or training regardless of energy level. An example quote is: “*… I go [training] without thinking about whether I have the energy or not; I just go”*.

### Qualitative Discussion

The qualitative analyses display experiences of individuals' intervention journeys. All *enabling talk* extracts come from pre-interviews with participants who, at follow-up, reported successfully maintaining behavior change. However, the content of the *enabling talk* theme can be utilized to detect how those who successfully maintained intervention-induced behavior change already resonate before the intervention. Consequently, they understand more about true motivation, expectations, efficacy, and future aims, leading to intervention-induced maintained change. Focus areas before and during the intervention that have been shown to have a positive impact on immediate and maintained behavior change are: having a specific target, having a positive attitude to physical activity, seeing change as a determination issue, engaging in positive self-talk, focusing on gains, and planning for the future. These themes could be developed and supported before and during the intervention to help each individual succeed in intervention-induced maintained behavior change. During and after intervention, focus areas to be supported are: not cheating during the intervention, paying attention to experiences of gains with the new behavior, continuing to view change as a determination issue, enjoying physical activity, planning for the future, and using intervention-induced positive impacts for further and continuous motivation to maintain the desired behavior. See Table 6 for a summary of the qualitative results.

**Table 6:** Summary of qualitative results.

**Pre-interviews**	**Target**	**Physical activity**	**Change approach**	**Self-talk**	**Motives**	**Future**
Disabling vs. enabling talk	Vague vs. clear	Dislike vs. positive attitude	Focus on difficulty vs. determination	Doubtful vs. positive	Avoidance vs. gain	Hinders vs. plan for maintenance
Follow-up interviews	Implementation	Physical activity	Change approach	Experience	Motivation	Future
Maintenance factors	No cheating	Enjoyment	Continuous determination	Focus on positive self-talk and gains	Utilizing positive impacts from new behavior	Planning for maintenance

## General Discussion

The aim of the present study was to further the understanding of participants' intervention journey to find helpful ways of supporting intervention-induced maintained behavior change. This was done by asking the two following questions: (1) Do levels of expectations and motivational factors predict immediate and maintained change? (2) How do intervention participants' perceptions describe their expectations, efficacy, and motivation in relation to intervention-induced immediate and maintained behavior change?

On an overall level, the study produced three main results. Firstly, the quantitative data showed that the pre-measurements of goal expectancy, efficacy, and motivation levels correlate with each other, but did not predict either immediate or maintained behavior change. Significant correlations were found between goal achievement (but not BMI change) and maintained behavior change. Secondly, the qualitative data showed that *enabling talk* was salient in the pre-interviews, with participants reporting successful immediate (and maintained) change. By contrast, pre-interview *disabling talk* turned out to be evident in interviews, with participants not responding to follow-up. Thirdly, when the qualitative and quantitative results are summarized and integrated, it appears that subjective goal achievement, in combination with enabling self-talk, are crucial factors for successfully maintaining behavior change.

Although aspects seen in previous research (e.g., Greaves et al., 2011)—such as behavior change models, processes leading to participation in interventions, and types of components that interventions should include—are important, the present study broadens the understanding of participant experiences linked with successful intervention-induced maintained behavior change.

In line with previous research (Anderson et al., 2001; Curioni and Lourenço, 2005), the results from the qualitative data show that most participants in present study succeeded in (immediate) behavior change during the intervention, resulting in weight loss. However, fewer succeeded in maintaining the intervention-induced behavior change or weight. It is noteworthy that while the motivational levels reported prior to the intervention correlated as expected, they did not predict either immediate or maintained change. While this is most likely a ceiling effect, given that participants who enroll in an intervention are highly motivated, it still indicates that levels of motivation, expectation, and efficacy alone are insufficient for understanding how to support each individual's intervention journey to maintain behavioral change.

Looking at the qualitative data, the results from the pre- and post-interviews show that participants who successfully maintained their behavior change engaged in *enabling talk*, whereas participants who did not maintain change engaged in *disabling talk*. This result shows that focusing on participants' experiences of interventions and their self-talk is a better way of supporting them in maintaining behavior change than their levels of motivation, expectation, and efficacy.

Interpreted together, the quantitative and qualitative data show the importance of setting attainable behavior goals in combination with enabling self-talk in order to maintain intervention induced behavior change (see Table 7 for a summary of the key findings, divided into quantitative and qualitative results). With the qualitative analysis results in hand, the question raised by the quantitative analysis results was how to understand that the pre-measurements are linked with each other as expected, but cannot explain either immediate or maintained change. The findings indicate that an answer might be found in deeper layers of the components that explain maintained change. The qualitative analysis results clarify that the quantitative pre-measurements do not explain immediate or maintained change because they do not capture each individual's actual motivation, expectation, or efficacy. Rather, the quantitative levels of motivation, expectation, and efficacy represent wishes for desired outcomes.

**Table 7:** Summary of key findings.

	**QUANT results (questionnaires + body measurements)**	**QUAL results (face-to-face interviews)**
Pre-measurements	Correlations: Expectation, efficacy, and motivation levels correlate with each other, but not with post or follow-up measures	Themes: Enabling vs. disabling pre-talk about target, physical activity, change approach, self-talk, motives, future when exposed to the concepts of expectation, efficacy, and motivation
Post-measurements	Correlations: Motivation type—goal achievement BMI change—goal achievement Goal achievement—maintained change *T*-test: Achievement of physical activity goals and eating behavior goals are more important for maintaining change than weight goals and other goals	Clarify that goal orientation during the intervention leads to maintained change Enabling pre-interview talk turned out to be extracted from interviews with participants reporting successful immediate (and maintained change). Disabling pre-interview talk turned out to be extracted from interviews with participants not responding to follow-up
Follow-up measurements	Correlations: Goal achievement—maintained change	Themes associated with maintained change: Not cheating during intervention Paying attention to gains with the new behavior Continuing to see change as a determination issue Enjoying physical activity Planning for the future Using the achieved results for further motivation to maintain the desired behavior

In line with Deci and Ryan (2008), the level of motivation does not correlate with goal achievement, but the motivation type does. Motivation level is the quantity of motivation (high to low), while motivation type is a description of the kind of motivation and what it consists of. All of the participants in the present study reported rather high levels (in both quantitative and qualitative data collection) of motivation, expectancy, and efficacy. While high-level reports throughout may generate a ceiling effect, the high-level reports of the present study might equally be a matter of cognitive dissonance (Festinger, 1957). In short, cognitive dissonance is the urge to behave in line with identified beliefs and attitudes. It would be illogical to invest money to join a behavior change intervention and, at the same time, report a low level of motivation to change. In the *disabling talk* category, high motivation level reports are not reflected in the same participants' motivation type reports. On the contrary, while they claim to have a high motivation level, they also display disabling talk regarding implementation describing avoidance, hindrances, and negative thoughts. Thus, from the perspective of cognitive dissonance, reports of high-level motivation by participants from the *disabling talk* category (which did not respond to follow-up) strive to maintain congruence between attitude (wanting to change) and action (signing up for a change intervention) rather than a fair picture of the behavior change commitment needed to successfully maintain behavior change. The explanation of striving to uphold congruence between attitude and action is supported by noteworthy differences regarding talk about motivation and goals between the *enabling talk* category and the *disabling talk* category. The *disabling talk* reported outcome goal focus (such as losing weight: *having* a lighter body), while the *enabling talk* reported emphasized behavior change goals (for example, *do* new activities, which will lead to a lighter body). Having set a behavior change goal, it seems more natural to be able to describe more details about the motivation; that is, why this is a good time to start and why the goals will be attained. When one has an outcome goal, the urge is to *have* rather than to *do*, which is why it is more problematic to define a detailed description of motivation to change behavior.

The same reasoning seems to be true regarding the overall high-level reports of goal expectancy and efficacy. Just like motivation level, these data were collected right before the start of the intervention. At this point, the participants had just invested their time, money, and energy in the intervention (action), and therefore they *should* have high expectations about reaching their goals and a high level of belief in themselves and the intervention (attitude). When looking at noteworthy differences in talk about future and change between the *enabling talk* category and the *disabling talk* category the real relationships between attitude and action are revealed. The overall high-level reports of goal expectancy and efficacy in the *disabling talk* category are contradicted in their talk about change and future, consisting of doubt, problems, difficulties, and hindrances for changing and maintaining change. These are obviously not characteristics of high expectancy, as compared to the *enabling talk* on change and future, which refers to change as creating routines and that change is about determination, including plans for how to maintain changed behaviors and how to tackle upcoming obstacles. Likewise, characteristics of high efficacy are trust and belief in self, but the *disabling talk* category was characterized by negative self-talk. This reveals a picture of actual low efficacy when complementing straight figures of level reports with story-telling narrative data.

The importance of making a distinction between level and type is prominent in SDT regarding motivation. The present study supports this theory when investigating motivation, and adds that the same is true regarding expectation and efficacy levels and types. Therefore, it is important to include types rather than solely high or low levels to build an understanding of actual motivation, expectancy, and efficacy, to enable optimized support in intervention-induced maintained change. However, instead of focusing only on motivation, expectancy, and efficacy levels, the results of this study suggest that it is better to focus on change approach, goals, future, self-talk, and motivation type, which give a more extensive representation of important factors for intervention-induced behavior change and its maintenance. It is better to focus on the individuals' narrative regarding the possibility to attain goals than more abstract notions of motivation.

Previous research has highlighted the importance of distinguishing between outcome goals and behavior change goals (Dweck, 1986), as well as avoidance vs. approach goals (Carver and Scheier, 1998). The present study supports the importance of making these distinctions and clarifies that outcome goals often are associated with avoidance motivation, while behavior change goals are associated with approach motivation. Further, outcome goals, as a set weight, are easy to measure and have a clear reaching point. Behavior change goals are more complex since they do not have a clear end point. It is satisfying to tick a box when something is completed, but behavior change is about continuity and can easily be measured in terms of the number of activities or experienced goal achievement. Hence, it is better to avoid avoidance motivation prior to approach motivation and to make continuous boxes to tick, both in order to monitor progress and accomplishment and to maintain the intervention-induced behavior and its gains.

In short, the results of the integrated qualitative and quantitative analysis show that individuals who focus on attainable behavior goals and whose narrative regarding themselves contain *enabling talk* are more likely to sustain an intervention-induced changed behavior.

From a practical perspective, the results of the present study can be utilized to help intervention participants succeed in intervention-induced maintained behavior change by early actions and resources invested at the right time and place. This can be achieved by firstly focusing on the actual behavior changes and setting behavior-related goals, rather than focusing on overall outcomes of the intervention. Secondly, it can be done by building personalized action plans and support based on individual needs and preferences. This can be done by focusing on the *enabling talk* themes before and during the intervention and the *follow-up* themes during and after the intervention.

### Limitations and Future Recommendations

With the high ecological validity benefits of a field study design, where no manipulations whatsoever are made to fit the study, the participants are in a natural environment (that is, intervention program), although there are also less favorable aspects. These include reduced control of extraneous variables and, in this case, fewer participants (for example, due to difficulties adding more participants to the intervention) and different samples of participants for the quantitative and qualitative aspects of the study. However, utilizing the quantitative data to suggest quantitative relationships and interpreting the quantitative data together with qualitative data helps to cover such a potential lack of power and create valuable synergy. Additionally, the participants in the qualitative data collection add to the total number of study participants. Another product of the field study origin is that the age span of the participants is limited to include more middle-aged participants. However, the ages ranged from 28 to 70 years.

We chose single-item measures for the quantitative aspect of the study, which could be compared to multi-item measures with potential benefits in reliability or capturing more information. However, we made this choice based on the benefits of single items' practicality and avoiding common methods bias (see, for instance, Bergkvist and Rossiter, 2007). Our choice of self-reported measures could also be a limitation. We compensated for that choice by combining self-reported measures with objective measures like body weight and BMI. However, objective measures could also be limitations. Maintained change is measured by body weight, which is an outcome rather than an actual behavior change. However, keeping a lower body weight (or continuing to lose weight) requires a change in either or both physical activity and eating behaviors, which is why we chose this objective measurement together with interview data, rather than solely a subjective self-report of behavior change.

Previous research (Ulen, 2008; Nordmo et al., 2020) has shown that weight loss usually peaks close to the intervention, before relapsing within a few years. Therefore, the follow-up in the present study—6 months after the intervention—might be quite early. However, while we have shown what happens before, during, and after the intervention with established maintenance measurements, future research may indicate what will happen years into the future. Further research is needed on the pre-eminent ways to enable genuine positive self-talk that maintains. However, the most comprehensive suggestion, to increase the possibility of maintaining intervention-induced behavior change, is to stimulate an enabling mindset (including seeing that change really *is* about determination).

Participation in the intervention was based on a monetary cost. On one hand, this leads to study participants being autonomously motivated to the degree that they invest their own money and time in an intervention without being given any incentives whatsoever. On the other hand, participants with a low income are likely to be less represented in the sample. However, the monetary cost was large compared to a regular gym membership including gym entrance only, but still an amount most people with priority could save up for in some months despite rather low income.

Lastly, collecting more demographic information about the participants would have been preferable for present study. And for future studies could interviewing the personal trainers be a way of capturing an additional perspective of the intervention-induced behavior change and course of events.

## Conclusion

Levels of expectations and motivational factors cannot be used alone to predict intervention-induced maintained change, but interpreting those results together with participants reasoning before the intervention can build an understanding of the intervention journey to form a guide for further directions on support and focus for successful maintained change.

To help improve successful intervention-induced maintained change, our results suggest that instead of focusing too much on increasing levels of expectancy, efficacy, and motivation prior to an intervention, it is better to identify and focus on strengthening each participant's individual weak spots. This includes individual support in structuring effective goal setting and future plan making, as well as a constructive approach toward key issues including monitoring goal attainment. More specifically, steps toward practically applying these findings include helping participants with the infrastructure to create clear behavior change goals (as compared to outcome goals) with detailed intervention plans, including support in organizing post-intervention plans. Working with approach and attitude toward the intervention-induced behaviors, change, and self are further actions toward successful maintained change. For instance, helping participants find physical activities that fit their personal preferences is probably the best way of supporting participants to create or increase a positive attitude to, and enjoyment of, physical activity.

## Data Availability Statement

The raw data supporting the conclusions of this article will be made available by the authors, without undue reservation.

## Ethics Statement

Ethical review and approval was not required for the study on human participants in accordance with the local legislation and institutional requirements. The patients/participants provided their written informed consent to participate in this study.

## Author Contributions

FS contributed to the data collection, data analysis, and manuscript writing. EW contributed to the data collection, analysis and interpretation of the results, and critical reviews of the manuscript. HG contributed to the analysis and interpretation of the results, and critical reviews of the manuscript. All three authors have contributed to the article and approved the submitted version.

## Conflict of Interest

The authors declare that the research was conducted in the absence of any commercial or financial relationships that could be construed as a potential conflict of interest.
